# Proton‐free induction decay MRSI at 7 T in the human brain using an egg‐shaped modified rosette K‐space trajectory

**DOI:** 10.1002/mrm.30368

**Published:** 2024-11-20

**Authors:** Simon Blömer, Lukas Hingerl, Małgorzata Marjańska, Wolfgang Bogner, Stanislav Motyka, Gilbert Hangel, Antoine Klauser, Ovidiu C. Andronesi, Bernhard Strasser

**Affiliations:** ^1^ German Center for Neurodegenerative Diseases (DZNE) Bonn Germany; ^2^ High Field MR Centre, Department of Biomedical Imaging and Image‐guided Therapy Medical University Vienna Vienna Austria; ^3^ Center for Magnetic Resonance Research, Department of Radiology University of Minnesota Minneapolis Minnesota USA; ^4^ Christian Doppler Laboratory for Clinical Molecular MR Imaging Medical University Vienna Vienna Austria; ^5^ Department of Neurosurgery Medical University of Vienna Vienna Austria; ^6^ Advanced Clinical Imaging Technology Siemens Healthcare AG Lausanne Switzerland; ^7^ A. A. Martinos Center for Biomedical Imaging, Department of Radiology Massachusetts General Hospital Charlestown Massachusetts USA; ^8^ Harvard Medical School Boston Massachusetts USA

**Keywords:** 7 T, gradient hardware restrictions, magnetic resonance spectroscopic imaging, modified rosette trajectory, non‐Cartesian trajectory, SNR efficiency

## Abstract

**Purpose:**

Proton (^1^H)‐MRSI via spatial‐spectral encoding poses high demands on gradient hardware at ultra‐high fields and high‐resolutions. Rosette trajectories help alleviate these problems, but at reduced SNR‐efficiency because of their k‐space densities not matching any desired k‐space filter. We propose modified rosette trajectories, which more closely match a Hamming filter, and thereby improve SNR performance while still staying within gradient hardware limitations and without prolonging scan time.

**Methods:**

Analytical and synthetic simulations were validated with phantom and in vivo measurements at 7 T. The rosette and modified rosette trajectories were measured in five healthy volunteers in 6 min in a 2D slice in the brain. An elliptical phase‐encoding sequence was measured in one volunteer in 22 min, and a 3D sequence was measured in one volunteer within 19 min. The SNR per‐unit‐time, linewidth, Cramer‐Rao lower bounds (CRLBs), lipid contamination, and data quality of the proposed modified rosette trajectory were compared to the rosette trajectory.

**Results:**

Using the modified rosette trajectories, an improved k‐space weighting function was achieved resulting in an SNR per‐unit‐time increase of up to 12% compared to rosette's and 23% compared to elliptical phase‐encoding, dependent on the two additional trajectory parameters. Similar results were achieved for the theoretical SNR calculation based on k‐space densities, as well as when using the pseudo‐replica method for simulated, in vivo, and phantom data. The CRLBs of γ‐aminobutyric acid and N‐acetylaspartylglutamate improved non‐significantly for the modified rosette trajectory, whereas the linewidths and lipid contamination remained similar.

**Conclusion:**

By optimizing the shape of the rosette trajectory, the modified rosette trajectories achieved higher SNR per‐unit‐time and enhanced data quality at the same scan time.

## 1 INTRODUCTION

Proton (^1^H)‐MR spectroscopic imaging (MRSI) allows for non‐invasive imaging of concentration distributions of the major metabolites in the human brain for (bio)medical applications.^1^, ^2^ MRSI studies at ultra‐high field (i.e., ≥7 T) benefit from increased spectral and spatial resolution, as well as an increase in the SNR.^3^, ^4^, ^5^, ^6^ The most common encoding strategy for MRSI is Cartesian phase encoding. However, this approach suffers from long acquisition times (TAs)^6^, ^7^, ^8^ because sampling only along the time‐dimension for each k‐space point is slow. In contrast, sampling along several k‐space dimensions and the time‐dimension simultaneously via spatial‐spectral encoding (SSE) accelerates data acquisition.^2^, ^9^ Several SSE techniques have been proposed such as echo‐planar spectroscopic imaging (EPSI),^10^, ^11^ spiral‐based trajectories,^12^, ^13^ rosette spectroscopic imaging (RSI),^14^, ^15^, ^16^, ^17^ and concentric ring trajectories (CRT).^18^, ^19^, ^20^, ^21^, ^22^, ^23^ Of these, self‐rewinding trajectories such as rosettes and CRT benefit from higher SNR‐efficiency at high spatial resolutions and spectral bandwidths because of not needing a rewinding gradient, and therefore, having no acquisition dead time during each circumnavigation of the trajectory. However, they need prewinder and rewinder gradient at the beginning and end of all circumnavigations, therefore, limiting the minimum TE and TR. Hingerl et al.^21^ showed that using a CRT readout at 7 T, eight major neurometabolites could be mapped with high spatial resolution over the majority of the brain with attractive TAs of 3 to 15 min. However, for even higher spatial resolutions or spectral bandwidths, CRT exceeds the limitations of current gradient systems.^24^, ^25^ Rosette trajectories are self‐rewinding, non‐Cartesian alternatives with lower hardware requirements,^14^ typically implemented via rosette petals that are distributed around the k‐space center. This reduces the maximum petal radius and hence, the required gradient slew rate by half compared to CRT.^14^ The reduced gradient stress makes rosette trajectories suitable for application at ultra‐high fields and very high spatial resolutions. However, this comes at the cost of an increased TA. To overcome this compressed sensing (CS) techniques have been implemented together with a 3D modified rosette trajectory, benefitting from its incoherent sampling pattern.^26^, ^27^, ^28^ Compared to density‐weighted‐CRT^19^, ^20^ the rosette trajectory has a reduced SNR efficiency because of unfavorable k‐space weighting. Kasper et al.^29^ showed that SNR can be maximized if no k‐space density compensation needs to be performed because the k‐space weighting function of a trajectory already matches the desired target weighting (density‐weighting). In addition to this SNR improvement, no density compensation during reconstruction is necessary.^19^ The increase in SNR is not the result of a difference in the nominal resolution of two k‐space weighting functions, but only the discrepancy between the measured and target k‐space densities.^29^, ^30^, ^31^, ^32^, ^33^ Because ^1^H‐MRSI is often strongly affected by lipid and other nuisance signals, a Hamming weighted k‐space is a desirable density function to reduce signal leakage.^20^ Rosette trajectories deviate substantially from the desired Hamming k‐space weighting.

Therefore, we propose an egg‐shaped modified rosette trajectory similar to the teardrop trajectory^34^ that allows for a more SNR‐efficient sampling of k‐space by changing the shape of the rosette petals. Without any increase in TA and with an adjustable demand on the gradient system, modified rosette trajectories present an enhancement to rosettes and are well‐suited for the use at ultra‐high field strengths and high spatial resolutions.

## 2 METHOD

### 2.1 Trajectory design

In contrast to the rosette trajectory that samples the entire k‐space along a continuous trajectory, our modified rosette trajectory consists of n individual petals in k‐space with radius $k_{\max}/2$ and a time varying compression factor (t). It can be written as:

(1) k(t)=(kxky)=12kmax(1−sinω1t2sinω2t2e(t)sinω1t2cosω2t2)⋅R(α)ω1=ω2=14kmax(1+cosω1te(t)sinω1t)⋅R(α),

where $k_{\max}=N_{x}/\left(2\cdot \mathrm{FoV}\right)$, $\omega_{1}=\omega_{2}=\text{πSBW}/\mathrm{nTi}$ with $\omega_{1}$ and $\omega_{2}$ being frequencies of oscillation along the radial and angular directions, respectively, nTi is the number of temporal interleaves, and SBW the spectral bandwidth. Please note that for the modified rosette trajectory $\omega_{1}$ and $\omega_{2}$ must be equal. The factors ½ and ¼ are necessary to cover the whole k‐space extent from −*k*_max_/2 to +*k*_max_/2 when rotating the trajectory with the rotation matrix R(α). The time varying compression factor is described by

(2) $$e\left(t\right)=E_{1}-\left(E_{1}-E_{2}\right)f\left(t\right),$$

where $E_{1}$, $E_{2}$, are the boundary factors of the compression. f(t) is a function with a range of 0≤f(t)≤1 for 0≤t≤T, where T is the time it takes to traverse each individual petal, so that f(t=0)=f(t=T)=0 and f(t=T/2)=1. Here, f(t) was chosen as f(t)=sin(ξ·t/2), which results in petals with the boundary compression factors $E_{1}$ influencing the compression in the k‐space center and $E_{2}$ at the position $k\left(T/2\right)=k_{\max}$. ξ is the modulation frequency of the boundary compression factors and is equal to ω to fulfill the above conditions.

**FIGURE 1:**
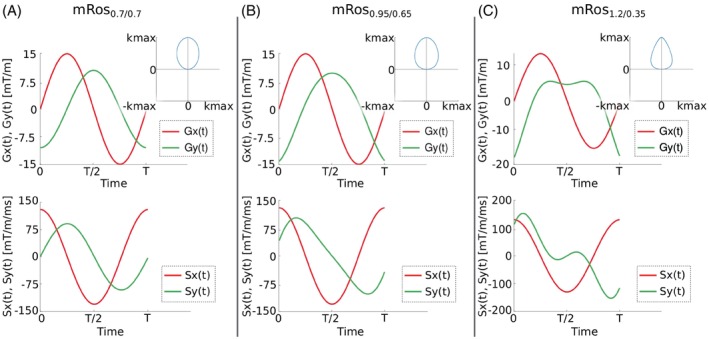
k‐Space trajectory, gradient amplitudes and slew rate of the modified rosette trajectory for a constant (A) and two sets of time‐dependent compression factors (B,C). The gradient moment and slew rate are shown for both phase encoding gradients for one period.

To acquire the entire k‐space, each individual petal is rotated around the k‐space center with varying angle α using the rotation matrix R(α) (Figure 1, Figure S1).

With varying boundary compression factors $E_{1}$, $E_{2}$, and function f(t) to transition between them, various petal shapes can be achieved. Using a time‐constant compression factor results in elliptical trajectories (Figure 1A). Figure 1B,C shows an “egg‐shaped” trajectory that maximizes SNR by best approximating Hamming weighting, while staying within the hardware limitations.

### 2.2 k‐Space density

**FIGURE 2:**
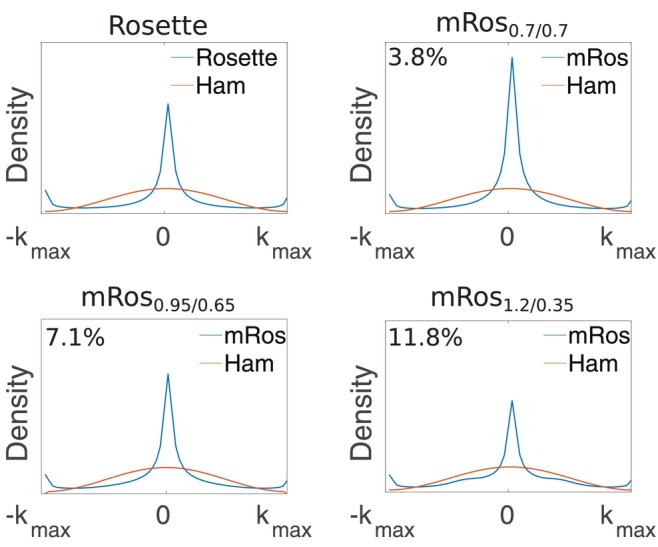
k‐Space density function of the rosette and modified rosette trajectories for a one‐dimensional line with *k*_y_ = 0 going through the k‐space center. The k‐space densities of the rosette, mRos_0.7/0.7_, mRos_0.95/0.65_, and mRos_1.2/0.35_ trajectories are shown. The relative SNR of the modified rosette trajectories to the rosette trajectory is written on the top left of each subfigure.

Using the two boundary compression factors, the oversampling of the rosette trajectory in the k‐space center and k‐space periphery can be reduced. This increases the SNR‐efficiency since it matches more closely the desired density of a Hamming function. Figure 2 shows the k‐space density functions of the rosette and modified rosette trajectories for a 1D line going through the k‐space center. Boundary compression factors >1 in the k‐space center reduce oversampling because larger circle radii result in a smaller density of sampling points. In the k‐space periphery, the desired lower k‐space weighting can be achieved using small circle radii. Here, a boundary compression factor <1 should be used.

### 2.3 Gradient amplitude and slew rate

The function e(t) also influences the gradient amplitude and slew rate. For a given spatial resolution and spectral bandwidth, the gradient amplitude and slew rate are minimized if a constant e(t)=1 is applied. For non‐constant e(t), they strongly depend on the difference $E_{1}-E_{2}$. The boundary compression factors were optimized for highest SNRs with the help of the simulations described in the following sections while staying within the gradient hardware restrictions. For the measurement parameters described later, the resulting trajectories had maximum gradient amplitudes of 14.9 mT/m, 14.9 mT/m, 15.1 mT/m, and 17.8 mT/m for the rosette trajectory, and modified rosette trajectories with three different sets of boundary compression factors ($E_{1}$/$E_{2}$=0.7/0.7, $E_{1}$/$E_{2}$=0.95/0.65, and $E_{1}$/$E_{2}$=1.2/0.35 referred to as mRos_0.7/0.7_, mRos_0.95/0.65_, and mRos_1.2/0.35_), respectively. The maximum gradient slew rates for the same trajectories were 130.1, 130.1, 143.9, and 191.9 mT/ms/m, respectively. All trajectories were calculated for the same parameters such as spectral bandwidth, number of angular and temporal interleaves, spatial resolution (see point 3.5 for the exact values).

### 2.4 Simulations

Kasper et al.^29^ showed that the SNR of a measurement is maximized when the k‐space density function matches the desired target function. Therefore, when choosing a Hamming filter, the SNR‐efficiency of a trajectory can be calculated via the SNR ratio to a hypothetical Hamming filter:

(3) $$\frac{{\mathrm{SNR}}_{\text{Acq}}}{{\mathrm{SNR}}_{\text{Hamming}}}=\sqrt{\frac{\sigma_{\text{Hamming}}^{2}}{\sigma_{\text{Acq}}^{2}},}$$

where $\sigma_{\text{Hamming}}^{2}$, $\sigma_{\mathrm{Acq}}^{2}$ are the noise variance of the Hamming and acquired k‐space weighting. The noise variance was calculated from the k‐space density function simulated using the acquisition parameters.^29^ We calculated the percentage deviation of the SNR of the modified rosette trajectories to the rosette trajectory, as well as to phase encoding and EPSI with ramp sampling. In addition to this analytical approach, the SNR was evaluated by simulating artificial k‐spaces using the reconstruction matrix from the in vivo measurements and applying the pseudo multiple replica‐based SNR calculation^35^ after reconstruction (see point 3.6 for more information). Here, the percentage deviation of the SNR of the modified rosette trajectories to the rosette trajectory was calculated.

### 2.5 Experiments

Data were acquired on a 7 T MAGNETOM+ (Siemens Healthineers AG) with a 32‐channel RF head coil for receive, and a volume coil for receive and transmit (Nova Medical) from a silicon oil and resolution phantom and seven healthy volunteers. Written informed consent was obtained from all volunteers. Three of the seven volunteers were measured twice.

In addition to the modified rosette trajectories with three different sets of boundary compression factors (mRos_0.7/0.7_, mRos_0.95/0.65_ and mRos_1.2/0.35_), the rosette trajectory was measured. The following parameters were used for both the phantom and in vivo measurements for all trajectories: matrix size 64 × 64, FOV 220 × 220 mm^2^, slice thickness 10 mm, resolution 3.4 × 3.4 × 10 mm^3^, two temporal interleaves, 840 FID points, spectral bandwidth 2778 Hz, ADC bandwidth 666.666 kHz (considering oversampling), and oversampling factor 2. All of the compared trajectories share the same trajectory parameters (i.e., 101 petals, 72 gradient points per petal, 240 ADC points per petal, k_max_ 0.145 mm^−1^, spectral bandwidth 2778 Hz). Two in vivo scans were obtained, one with WET water suppression, four averages and TR 440 ms, TE 1.1 ms, TA 6:04 min:s, and the other without water suppression, one average and TA 1:31 min:s for estimating the coil combination weights. The latter settings were also used for the phantom measurements. Five volunteer were measured for these comparisons.

Five volunteers were measured using the mRos_1.2/0.35_ trajectory and an elliptical phase‐encoding sequence in an additional session. While the mRos_1.2/0.35_ trajectory was measured with the parameters described above the following parameters were used for the elliptical phase‐encoding sequence: matrix size 64 × 64, FOV 220 × 220 mm^2^, slice thickness 10 mm, resolution 3.4 × 3.4 × 10 mm^3^, 2048 FID points, spectral bandwidth 4200 Hz, and oversampling factor 2. Two in vivo scans were obtained, one with WET water suppression, one averages and TR 440 ms, TA 22:02 min:s, and the other without water suppression, one average and TA 2:30 min:s for estimating the coil combination weights.

The four trajectories were estimated based on oil phantom measurements using an adapted method of Brodsky et al.^36^ and Robison et al.^37^ by measuring the phase effect of each gradient using thin slices orthogonal to the investigated gradient with negative and positive slice offsets and negative and positive gradient polarities. We used the same parameters as for the in vivo measurements, except: slice thickness 1 mm, slice offsets of 20 mm, TE 6.02 ms, and using the volume coil for signal reception.

A 3D‐T_1_‐weighted magnetization prepared rapid gradient echo (MP2RAGE)^38^ sequence was measured in the in vivo scan protocol for anatomical reference with a nominal resolution of 1.1 × 1.1 × 1.2 mm^3^ and TA of 4:57 min:s. 3D FID‐MRSI scans using the mRos_1.2/0.35_ were measured, once with water suppression, and once without. The following parameters were used: matrix size 64 × 64 × 17, FOV 220 × 220 × 76 mm^3^, two temporal interleaves, 840 FID points, spectral bandwidth 2778 Hz, one average, TR 440 ms, and TA 18:44 min:s. Note that the acquisition of the coil combination weights can be highly accelerated, but this was not the purpose of this paper. Before the measurements, manual shimming was performed. MRSI data were filtered to a Hamming weighting in k‐space, and reconstructed with a type 2 non‐uniform discrete Fourier transform using the measured trajectories. A lipid removal using an L1‐regularization was performed.^39^

### 2.6 Evaluation

In vivo spectra were fitted using LCModel (Figure S2).^40^ Our basis set included 17 simulated brain metabolites and a measured macromolecular background.^41^ SNR per‐unit‐time ratios were calculated for all volunteers and trajectories using the pseudo replica method. The noise was estimated for each voxel using the SD through a stack of pseudo replicas of the true image with added, uniformly distributed, and correctly scaled noise. This is a simulation‐equivalent of measuring multiple times the same data, and was shown to provide good estimate for the SD of the noise.^29^ The signal was defined as the amplitude of the fitted NAA peak.^42^ A mean over all voxels inside a brain mask derived from the T_1_‐weighted images was calculated. The mean and standard error over all volunteers is reported. The same comparison is performed for the Cramer‐Rao lower bounds (CRLB) values of NAA, total creatine (tCr), total choline (tCho), glutamate (Glu), myo‐inositol (myo‐Ins), *N*‐acetylaspartylglutamate (NAAG), glutathion (GSH), and γ‐aminobutyric acid (GABA), the spectral linewidth, and the lipid SNR. The CRLBs and linewidths are calculated by LCModel, whereas the lipid SNR was calculated by summing the magnitude spectra in the range of 0.0 to 1.8 ppm and dividing by the SD of the noise. Metabolic maps and spectra are shown for the first volunteer in agreement with minimum reporting standards^43^ (Table S1).

Two‐sided paired *t* tests were performed to test for statistically significant differences between SNR per‐unit‐time, linewidth, CRLB, and lipid SNR values of the different modified rosette trajectories to the rosette trajectory.

## 3 RESULTS

### 3.1 SNR Simulations

The k‐space density simulations evaluate to an SNR increases of 3.8%, 7.1%, and 11.8% to the rosette trajectory for the mRos_0.7/0.7_, mRos_0.95/0.65_, and mRos_1.2/0.35_ trajectories, respectively. Compared to phase encoding the simulations showed significant SNR improvements of 16.6%, 20.19%, 24.5% and 32.01%, 36.10%, 42.08% compared to EPSI for the mRos_0.7/0.7_, mRos_0.95/0.65_, and mRos_1.2/0.35_ trajectories, respectively. Using the pseudo multiple replica method on simulated data, percentage increases compared to the rosette trajectory of 2.0%, 2.6%, and 8.7% for the mRos_0.7/0.7_, mRos_0.95/0.65_, and mRos_1.2/0.35_, respectively, were estimated.

### 3.2 Phantom results

**FIGURE 3:**
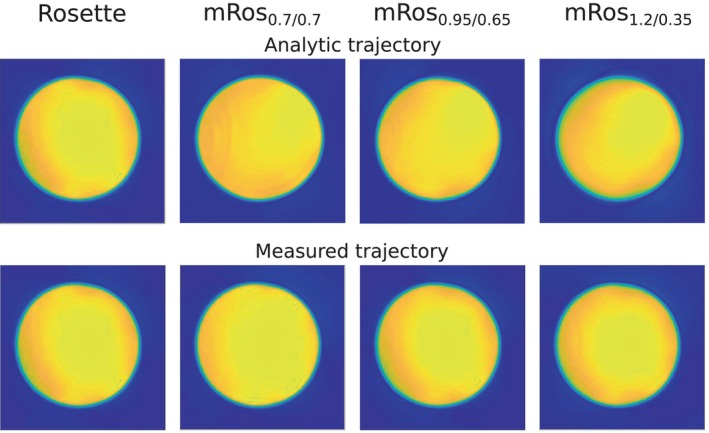
Results from the phantom measurement using a silicon oil phantom and the in vivo protocol (FOV of 220 × 220 mm^2^, slice thickness 10 mm, and matrix size of 64 × 64). The phantom was measured with the rosette, mRos_0.7/0.7_, mRos_0.95/0.65_, and mRos_1.2/0.35_ trajectories. The phantom results were Hamming weighted and zero‐filled to obtain a matrix size of 300 × 300. Images were reconstructed with an analytically determined (top row) and measured (bottom row) trajectories. Using a measured trajectory eliminated significant artifacts from gradient trajectory errors that can be observed for mRos_1.2/0.35_.

In Table S2 the calculated SNRs per‐unit‐time from phantom measurements are reported. For better comparison, the percentage deviation of the mean SNR per‐unit‐time values calculated over the entire phantom for the modified rosette trajectories to the rosette trajectory is shown. All three modified rosette trajectories showed significantly higher SNRs per‐unit‐time compared to the rosette trajectory. Over all three measurements the SNR per‐unit‐time increased in comparison to rosette trajectory by 3.81% ± 0.2% (*p* ≪ 0.001), 7.00% ± 0.17% (*p* ≪ 0.001), and 12.47% ± 0.29% (*p* ≪ 0.001) for the mRos_0.7/0.7_, mRos_0.95/0.65_, and mRos_1.2/0.35_ trajectories, respectively. In Figure 3, the reconstructed images from the phantom measurements are shown. No differences can be seen in the images reconstructed with the measured trajectories (Figure 3, bottom row). Using an analytically determined trajectory lead to localization errors near the edges of the phantom when the modified rosette trajectory was used (Figure 3, top row). In Figure S3, the results of the resolution phantom measurement are shown. Similar resolution properties can be observed for the rosette and modified rosette trajectories.

### 3.3 In vivo results

**FIGURE 4:**
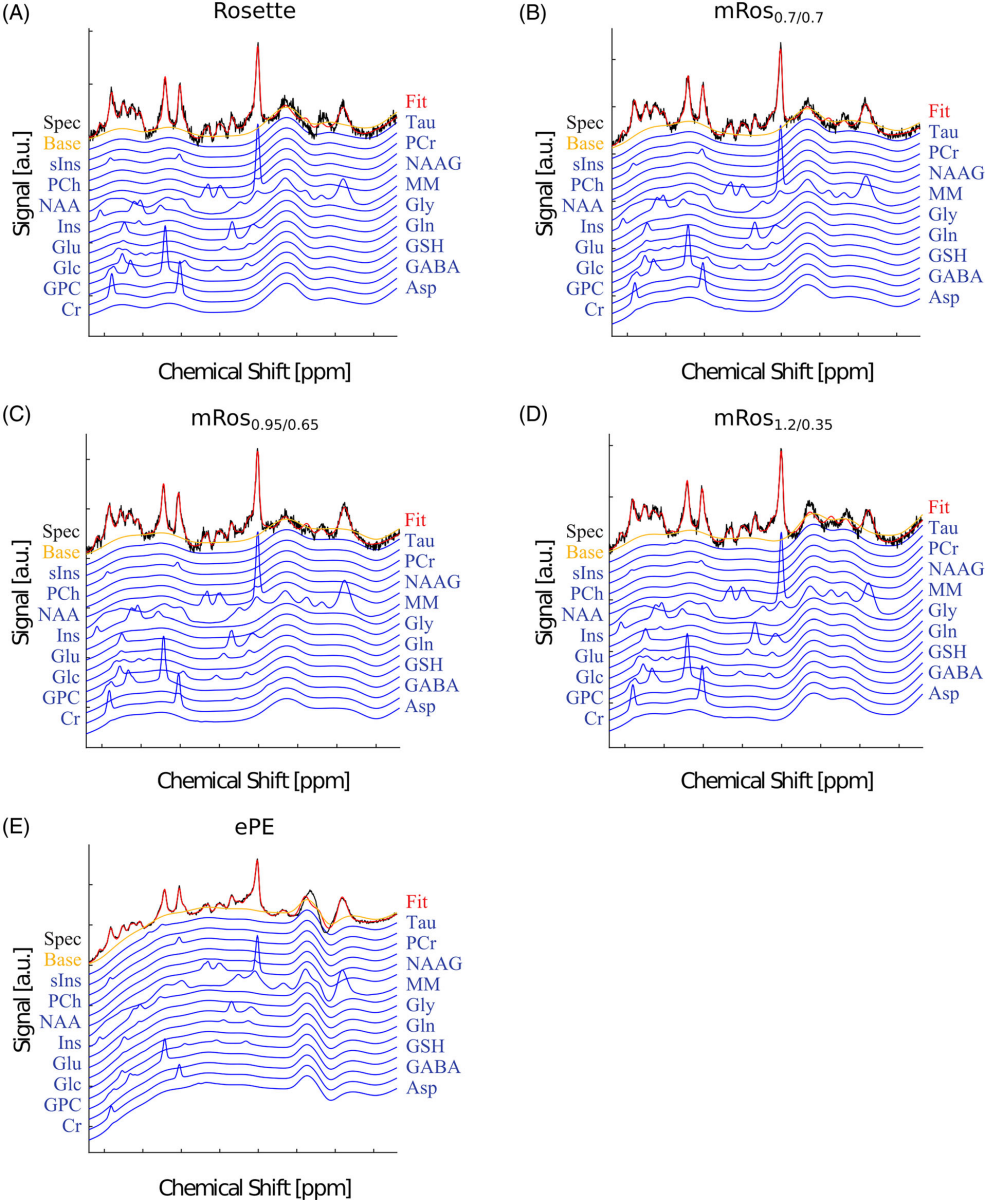
In vivo spectra from the first volunteer and all measured trajectories (rosette [A], mRos_0.7/0.7_ [B], mRos_0.95/0.65_ [C], mRos_1.2/0.35_ [D], and elliptical phase‐encoding [E]). Fitted spectra (red) and measured data (black) were taken from LCModel. First order phase correction was performed. Similar spectral noise can be observed for the spectra acquired with the rosette, mRos_0.7/0.7_, mRos_0.95/0.65_, and mRos_1.2/0.35_ trajectories. The voxel location from which the spectra are taken is shown in Figure 5.

**FIGURE 5:**
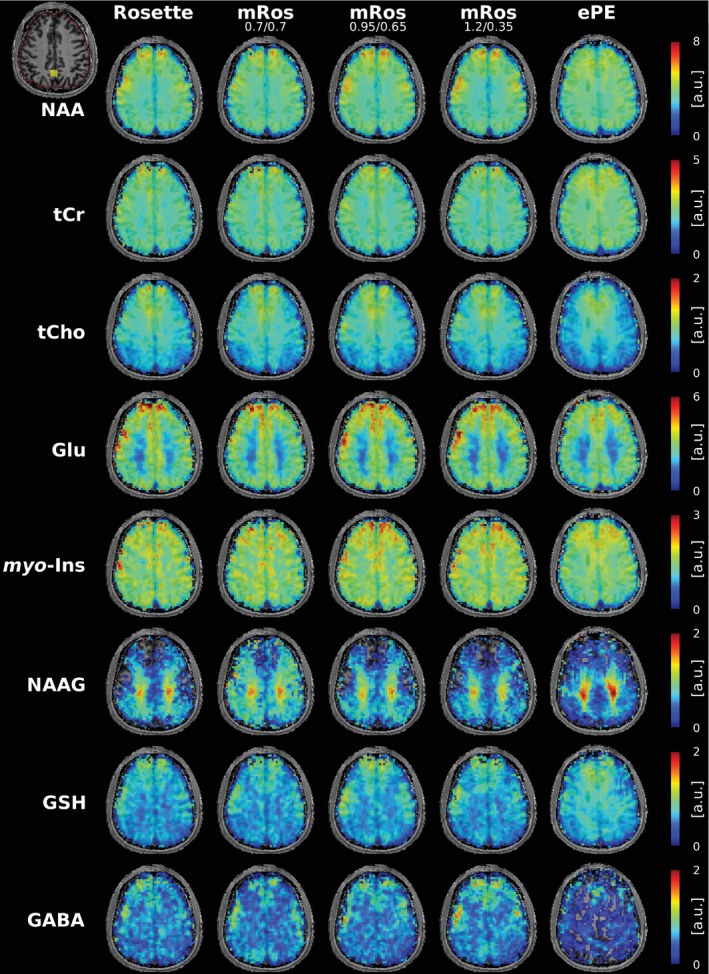
Results from the first volunteer using the rosette, mRos_0.7/0.7_, mRos_1.2/0.35_ and mRos_0.95/0.65_ trajectory and elliptical phase‐encoding (FOV of 220 × 220 mm^2^, slice thickness 10 mm and matrix size of 64 × 64). Metabolic maps for NAA, total creatine (tCr), total choline (tCho), glutamate (Glu), myo‐inositol (myo‐Ins), *N*‐acetylaspartylglutamate (NAAG), glutathion (GSH), and γ‐aminobutyric acid (GABA), are displayed. The region of interest (ROI) and voxel location of the spectra shown in Figure 4 is shown in the top left.

**FIGURE 6:**
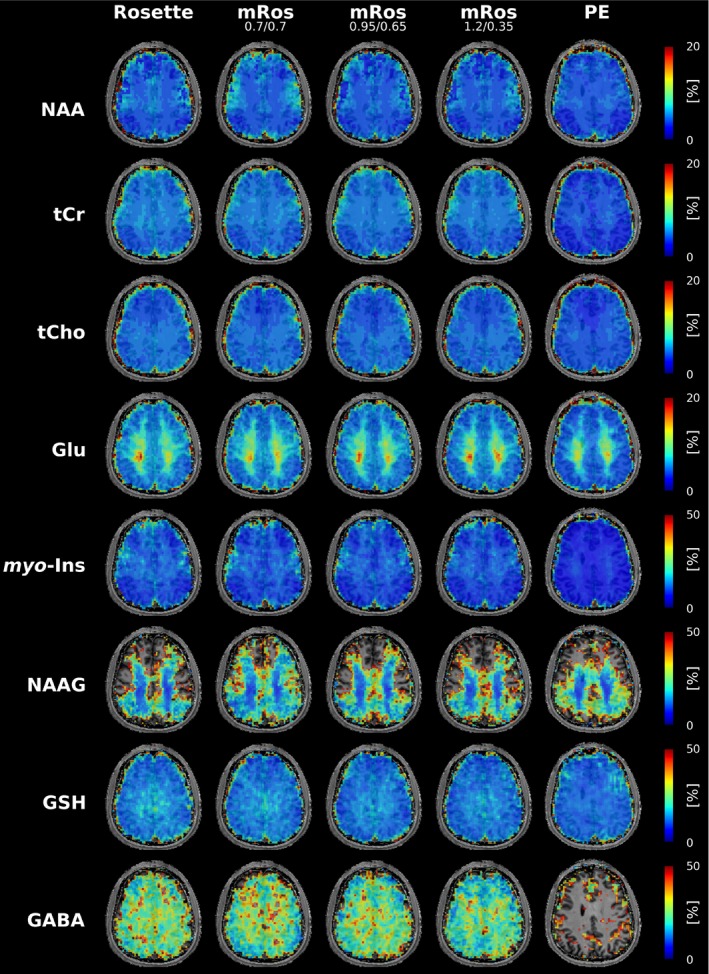
Cramer‐Rao lower bounds (CRLB) maps for the metabolites shown in Figure 5. CRLB values were taken from LCModel.

Table 1 shows the calculated SNR per‐unit‐time deviation of the modified rosette trajectories in comparison to the rosette trajectory. Mean SNR per‐unit‐time gains of 3.0% (*p* = 0.03), 6.3% ± 1.1% (*p* < 0.03), and 8.9% ± 1.6% (*p* < 0.03) can be observed over all subjects for the mRos_0.7/0.7_, mRos_0.95/0.65_, and mRos_1.2/0.35_ trajectories, respectively. Compared to elliptical phase‐encoding a mean SNR per‐unit‐time gain of 21.0% ± 4.8% (*p* < 0.03) was observed. Representative spectra for the first volunteer at a voxel location specified by the minimum reporting standards^43^ for all compared trajectories are shown in Figure 4. The raw spectra and fitted data are displayed. Good fitting results with similar noise can be observed in the spectra for all trajectories. Reduced noise can be observed in the spectrum measured with the elliptical phase encoding sequence. Two dimensional metabolic maps (Figure 5) and CRLB maps (Figure 6) for the first volunteer are shown. Hardly any differences between the trajectories are visible for NAA, tCr, tCho, Glu, myo‐Ins, and GSH. Using the mRos_1.2/0.35_ trajectory, improved GABA maps were achieved as a result of reduced CRLB values. For NAAG reduced CRLB values and improved maps were observed using the mRos_0.7/0.7_ trajectory. Some maps have more outliers in the front of the brain, which might stem from incorrect coil combination weights. Reduced CRLB values can be observed for the elliptical phase encoding sequence, leading to improved metabolic maps. GABA could not be fitted reliably for the elliptical phase‐encoding sequence. In the 3D metabolic (Figure 6) and CRLB (Figure S4) maps, similar fitting results were achieved for all metabolites except NAAG compared to the 2D acquisition. However, the 3D dataset had more ringing artifacts in some metabolic maps, which we believe was because of B_0_‐inhomogeneities. 2D SNR maps for the first volunteer are shown in Figure 8.

**TABLE 1:** Mean and standard error SNR per‐unit‐time calculated using the pseudo‐replica method over all voxels inside an ROI (see Figure 5) for all volunteers and the trajectories with different compression factors.

	Rosette	mRos_0.7/0.7_	mRos_0.95/0.65_	mRos_1.2/0.35_	mRos_1.2/0.35_	ePE
Vol 1	1.37 ± 0.34	1.42 ± 0.33	1.48 ± 0.34	1.53 ± 0.36	1.54 ± 0.01	1.26 ± 0.01
Vol 2	1.36 ± 0.34	1.41 ± 0.35	1.44 ± 0.36	1.49 ± 0.37	1.62 ± 0.01	1.28 ± 0.01
Vol 3	1.40 ± 0.33	1.44 ± 0.32	1.47 ± 0.33	1.51 ± 0.35	1.54 ± 0.01	1.25 ± 0.01
Vol 4	1.20 ± 0.32	1.26 ± 0.32	1.28 ± 0.32	1.31 ± 0.35	1.68 ± 0.01	1.46 ± 0.01
Vol 5	1.31 ± 0.34	1.31 ± 0.32	1.39 ± 0.35	1.40 ± 0.36	1.42 ± 0.01	1.22 ± 0.01
Mean	1.33 ± 0.08	1.37 ± 0.08	1.41 ± 0.08	1.45 ± 0.09	1.56 ± 0.1	1.29 ± 0.1

Abbreviations: ROI, region of interest; Ros, modified rosette.

**FIGURE 7:**
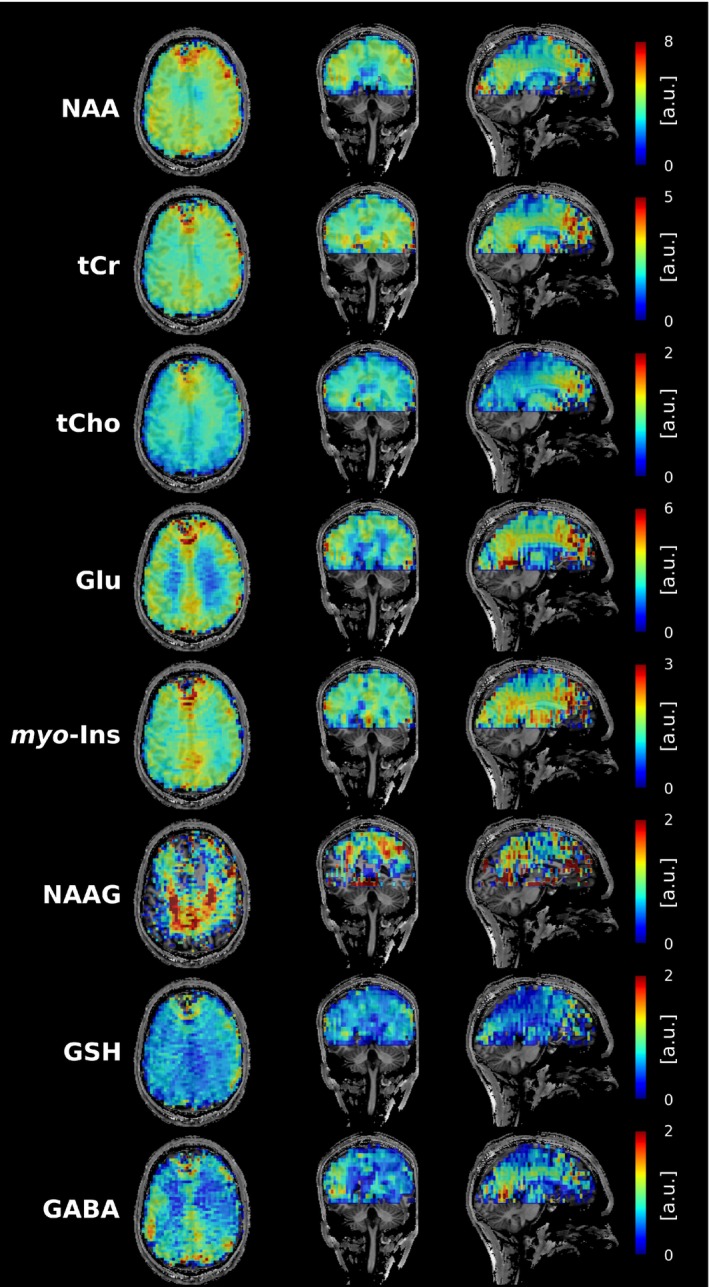
Whole‐brain metabolic maps acquired with the mRos_1.2/0.35_ trajectory. A matrix size of 64 × 64 × 17 was measured in 19 min.

**FIGURE 8:**
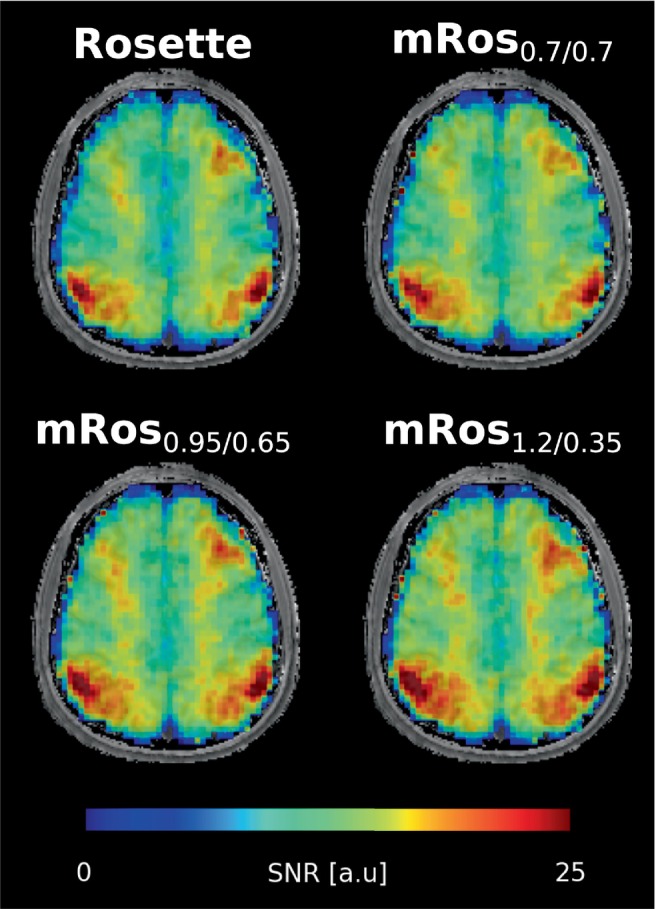
SNR maps from the first volunteer for the rosette and modified rosette trajectories.

**FIGURE 9:**
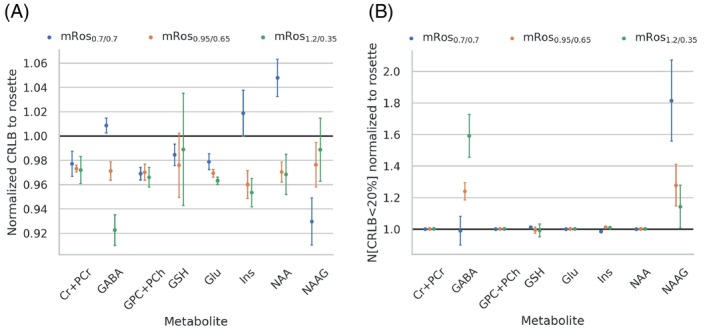
Mean of the Cramer‐Rao lower bounds (CRLBs) over all voxels inside the brain with CRLB <50% for NAA, total creatine (tCr), total choline (tCho), glutamate (Glu), myo‐inositol (myo‐Ins), *N*‐acetylaspartylglutamate (NAAG), glutathion (GSH), and γ‐aminobutyric acid (GABA). The number of voxels with CRLB <20% are shown for the same metabolites.

The mean CRLB values of all voxels with CRLB<50% for NAA, tCr, tCho, Glu myo‐Ins, NAAG, GSH, and GABA are shown in Figure 9. Most voxels inside the brain mask (Figure 5) had CRLBs <50% for all metabolites except NAAG. Similar means can be observed for the rosette and modified rosette trajectories for all metabolites except GABA and NAAG. Across all volunteers the mRos_1.2/0.35_ shows lower mean CRLBs than the other trajectories. Similarly, the mRos_0.7/0.7_ has lower mean CRLBs than the other trajectories for NAAG. An increase of the number of voxels with CRLB values below 20% is observed for the mRos_1.2/0.35_ for GABA and mRos_0.7/0.7_ for NAAG compared to the rosette trajectory. The linewidths were 14.24 ± 0.1 Hz, 14.25 ± 0.1 Hz, 14.27 ± 0.1 Hz, and 14.38 ± 0.1 Hz for the rosette, mRos_0.7/0.7_, mRos_0.95/0.65_, and mRos_1.2/0.35_ trajectories, respectively. The lipid SNR normalized to the rosette values were 1.00 ± 0.10, 0.79 ± 0.036, 1.16 ± 0.05, and 1.07 ± 0.05. Paired *t* tests between the values of the modified rosette trajectories and the rosette showed that the CRLB, linewidth, and lipid SNR are non‐significantly different. The mean CRLB values of the mRos_1.2/0.35_ trajectory normalized to elliptical phase‐encoding for NAA, tCr, tCho, Glu, myo‐Ins, NAAG, GSH, and GABA were 1.09 ± 0.02 (*p <* 0.02), 1.29 ± 0.02 (*p <* 0.01), 1.2 ± 0.02 (*p <* 0.02), 1.23 ± 0.03 (*p <* 0.02), 1.4 ± 0.03 (*p* < 0.01), 1.05 ± 0.04 (*p* = 0.4), 1 ± 0.01 (*p* = 0.7), and 1 ± 0.04 (*p* < 0.01), respectively. The linewidths were 14.42 ± 0.09 for mRos_1.2/0.35_ and 12.78 ± 0.09 for elliptical phase‐encoding (*p <* 0.03). The lipid SNR of the mRos_1.2/0.35_ trajectory normalized to elliptical phase‐encoding was 1.21 ± 0.02 (*p <* 0.03).

## 4 DISCUSSION

We have shown that by modifying the rosette trajectory higher SNR per‐unit‐time values of up to +12% can be achieved. The SNR per‐unit‐time results from phantom and in vivo measurements were consistent with simulations, but a smaller SNR per‐unit‐time increase was observed in the in vivo data using the mRos_1.2/0.35_ trajectory (Figure S5). The reason for that might be that this trajectory is more susceptible to subject related artifacts such as motion. The highest SNR per‐unit‐time was achieved with the mRos_1.2/0.35_ (˜9% higher than rosette), followed by the mRos_0.95/0.65_ (˜6% higher than rosette) and mRos_0.7/0.7_ (˜3% higher than rosette), whereas the lowest SNR per‐unit‐time was achieved with rosette trajectory. Because of the similar design of the trajectories and the same number of sampling points along the trajectory, no additional measurement time is necessary to realize this SNR per‐unit‐time gain. Compared to elliptical phase‐encoding the mRos_1.2/0.35_ achieved a ˜21% increase in SNR per‐unit‐time, which is consistent with the first simulation. All compared trajectories had similar linewidths, lipid contaminations, and resolution properties. The non‐significant decrease of the mean CRLBs for GABA of the modified rosette data in comparison to the rosette data indicate that the SNR per‐unit‐time increase leads to a small improvement in metabolite quantification precision for low‐concentration metabolites. The mRos_0.7/0.7_ trajectory showed improved NAAG maps and reduced CRLBs compared to the other trajectories, despite lower SNR per‐unit‐time than the other modified rosette trajectories. The reason for this remains unknown. The increase in measurement time of the elliptical phase encoding sequence lead to a reduction in most CRLBs and linewidths while the lipid SNR increased slightly.

Kasper et al.^29^ and several others showed that the sampling SNR‐efficiency is highest, when the measured k‐space density matches the desired k‐space density.^31^, ^32^, ^33^, ^44^ This can be understood intuitively: Measuring data with matched density focuses on important regions allowing to average more data there, and avoiding unnecessary noise from less important areas. In contrast, rescaling the data in postprocessing rescales signal and noise exactly the same. It is important to understand that with a “desired k‐space density” we mean to achieve this density in postprocessing by compensating any remaining discrepancies between the measured and desired density. Our desired k‐space density was a Hamming filter, which is reasonably well approximated by modified rosette trajectories, and less so by rosette trajectory. However, despite this closer approximation to Hamming weighting, density filtering is still required before reconstruction for all trajectories. Both, the rosette and modified rosette trajectories have mild oversampling of the k‐space center. This comes at the disadvantage of less efficient k‐space sampling and a greater deviation from the ideal k‐space weighting. However, mild oversampling of the k‐space center makes the trajectories more robust against subject motion as shown by Schirda et al.^14^

The slew rate of the mRos_1.2/0.35_ trajectory of 191.9 mT/ms/m was close to the hardware limit of 200 mT/ms/m and noticeably higher compared to that of the rosette and mRos_0.7/0.7_ trajectories, which used 130.1 mT/ms/m. Measurements with the mRos_0.95/0.65_ trajectory resulted in a ˜6% SNR per‐unit‐time increase. With maximum slew rates of 143.9 mT/ms/m, which are slightly higher than those of the rosette, the mRos_0.95/0.65_ trajectory can be a good compromise between high SNR per‐unit‐time and reduced gradient stress.

A major advantage of modified rosette trajectories is the flexibility to adjust the two compression factors so that a given slew rate restriction is met, whereas maximizing the SNR per‐unit‐time. By doing so, the sequence can be adapted for several different modalities, such as different magnetic field strengths or matrix sizes. The smallest achievable slew rate is that of the rosette trajectory. All compared trajectories provided similar spectra and metabolic maps, indicating comparable spectral qualities between all trajectories.

In comparison to other SSE trajectories, such as spiral and EPSI trajectories, modified rosette trajectories benefit from their self‐rewinding properties. This provides higher SNR because of not needing a rewinder gradient. Moreover, the k‐space density of modified rosette trajectories is closer to a Hamming filter than most other MRSI trajectories, except the ideally shaped density‐weighted CRT sampling.^19^, ^20^ Modified rosette trajectories achieve their k‐space density not by measuring additional angular interleaves as is the case for density‐weighted CRT, but by changing the gradient shape. Therefore, the modified rosette trajectories do not prolong the measurement while achieving a comparable SNR‐efficiency as density‐weighted CRT encoding. In addition, the modified rosette trajectories, as well as the rosette use smaller circles than CRT, and therefore, can cover much higher spectral bandwidths for a given resolution, which is essential for MRSI at ≥7 T^14^. For example, using the in vivo parameters (2778 Hz SBW, 3.4 mm in‐plane resolution, 2 temporal interleaves, TR 440 ms, maximum slew rate of 192 mT/m/ms) CRT or EPSI with ramp‐sampling is not possible. However, EPSI with ramp‐sampling can be done with a similar SBW of 2667 Hz, but the SNR‐efficiency in comparison to a Hamming filter drops to only 57% (for comparison: rosette: 79%, mRos_1.2/0.35_: 89%). The measurement time for the 3D protocol would be 15:57 min:s. CRT can only achieve similar parameters with three temporal interleaves, resulting in 8:56 min:s measurement time for the 3D protocol, and an SNR‐efficiency of 91.72%. However, at an in‐plane resolution of 1.7 mm or spectral bandwidth of 3840 Hz (e.g., for 9.4 T measurements), even three temporal interleaves are not enough for CRT, whereas it is still possible to measure the modified rosette trajectories with three temporal interleaves. Increasing the number of temporal interleaves even further is not feasible because it causes spectral artifacts.^20^ Modified rosette trajectories share the benefit of circular k‐space coverage of other non‐Cartesian SSE strategies such as spirals, rosettes and CRT. This results in a more time‐efficient k‐space acquisition for a given resolution, as including the corners of k‐space (e.g., like in EPSI) cause an anisotropic spatial resolution.^45^ Another advantage of modified rosette trajectories is that they pass through the k‐space center in each circumnavigation, which opens the possibility to correct for instabilities such as motion, frequency drifts or gradient delays without the need for additional measurements.^46^

### 4.1 Limitations

The gradient amplitudes and slew rates correlate with the compression factors of the modified rosette trajectories. At ultra‐high fields $B_{0}\geq$7 T this can lead to increased gradient hardware stress, which has to be well balanced. Especially beyond 10 T, where higher spectral bandwidths and spatial resolution are expected, this limits the possible SNR gain. Similarly, higher matrix sizes lead to increased slew rates. This could be mitigated by the use of temporal interleaves, which would, however, increase scan times and could—for ^1^H MRSI—potentially introduce unwanted spectral artifacts associated with unsuppressed water signals. In general, phantom measurements showed a higher susceptibility of modified rosette trajectories to gradient trajectory errors, if no compensation was used, leading to incorrect signal allocation in the reconstructed image.^47^ However, this problem was fully corrected for by using measured trajectories.

In general, modified rosette and rosette trajectories have longer TAs than SSE approaches like spirals, CRTs and EPSI. This makes them more vulnerable to motion artifacts and less clinically attractive. However, at high‐resolution, high‐bandwidth 3D coverage, these alternatives are not feasible because of gradient performance limitations. Shen et al.^26^, ^27^ showed that using 3D modified rosette trajectories together with CS only 40% of all rosette petals are needed for artifact free images. The possibility of accelerating modified rosette trajectories using CS reconstruction approaches proved useful to a variety of application in recent publications.^48^, ^49^, ^50^, ^51^, ^52^, ^53^, ^54^ Modified rosette trajectories will likely require a combination with complimentary acceleration methods such as parallel imaging or compressed sensing to reach clinically attractive scan times.

## 5 CONCLUSIONS

We have shown that high‐resolution metabolic mapping using modified rosette trajectories benefits from increased SNR efficiency, whereas staying within the gradient performance limits in comparison to other SSE approaches. Using the two compression parameters, trajectories can be adapted to a variety of applications. This makes rosettes especially interesting for MRSI at ultra‐high fields.

## CONFLICT OF INTEREST STATEMENT

A.K. is an employee of Siemens Healthineers International AG, Switzerland. The other authors have no conflicts of interest to declare.

## Supporting information


**Figure S1.** Distribution of petals in k‐space of the sequences compared (top row). In the bottom row the analytic and measured k‐space trajectory can be observed for the petal with rotation angle α=π (red box in the top row).
**Figure S2.** LCModel control file from an exemplary voxel.
**Figure S3.** Resolution phantom experiments with the in vivo protocol. Pixel intensities along a horizontal line in the center of the image are shown below. Comparable resolution capabilities can be observed for all compared trajectories.
**Figure S4.** Whole‐brain CRLB maps of the metabolites shown in Figure 7.
**Figure S5.** Boxplot showing the increase in SNR per‐unit‐time for the modified rosette trajectories compared to the rosette. The results of the phantom and in vivo measurements are shown.
**Table S1.** Minimum Reporting Standards for in vivo MR Spectroscopy Note. – Parameters 7 TDMI, CRLB = Cramér‐Rao lower bounds; FID = free induction decay; FOV = field of view; SNR = signal‐to‐noise ratio; VOI = volume of interest.
**Table S2.** Results of the SNR per‐unit‐time calculation for the rosette trajectory and modified rosette trajectories from the phantom measurement. SNR values are displayed as the percentage difference from the rosette trajectory. The mean and standard error over all three measurements are shown.
