# Supplementary material for: Proton‐free induction decay MRSI at 7 T in the human brain using an egg‐shaped modified rosette K‐space trajectory
Source: Magn Reson Med. 2024 Nov 20;93(4):1443–57. doi: 10.1002/mrm.30368 (PMC11782714; doi:10.1002/mrm.30368)
Supplement: Supplementary file 1 — Figure S1. Distribution of petals in k‐space of the sequences compared (top row). In the bottom row the analytic and measured k‐space trajectory can be observed for the petal with rotation angle α=π (red box in the top row). Figure S2. LCModel control file from an exemplary voxel. Figure S3. Resolution phantom experiments with the in vivo protocol. Pixel intensities along a horizontal line in the center of the image are shown below. Comparable resolution capabilities can be observed for all compared trajectories. Figure S4. Whole‐brain CRLB maps of the metabolites shown in Figure 7. Figure S5. Boxplot showing the increase in SNR per‐unit‐time for the modified rosette trajectories compared to the rosette. The results of the phantom and in vivo measurements are shown. Table S1. Minimum Reporting Standards for in vivo MR Spectroscopy Note. – Parameters 7 TDMI, CRLB = Cramér‐Rao lower bounds; FID = free induction decay; FOV = field of view; SNR = signal‐to‐noise ratio; VOI = volume of interest. Table S2. Results of the SNR per‐unit‐time calculation for the rosette trajectory and modified rosette trajectories from the phantom measurement. SNR values are displayed as the percentage difference from the rosette trajectory. The mean and standard error over all three measurements are shown. [file MRM-93-1443-s001.docx]

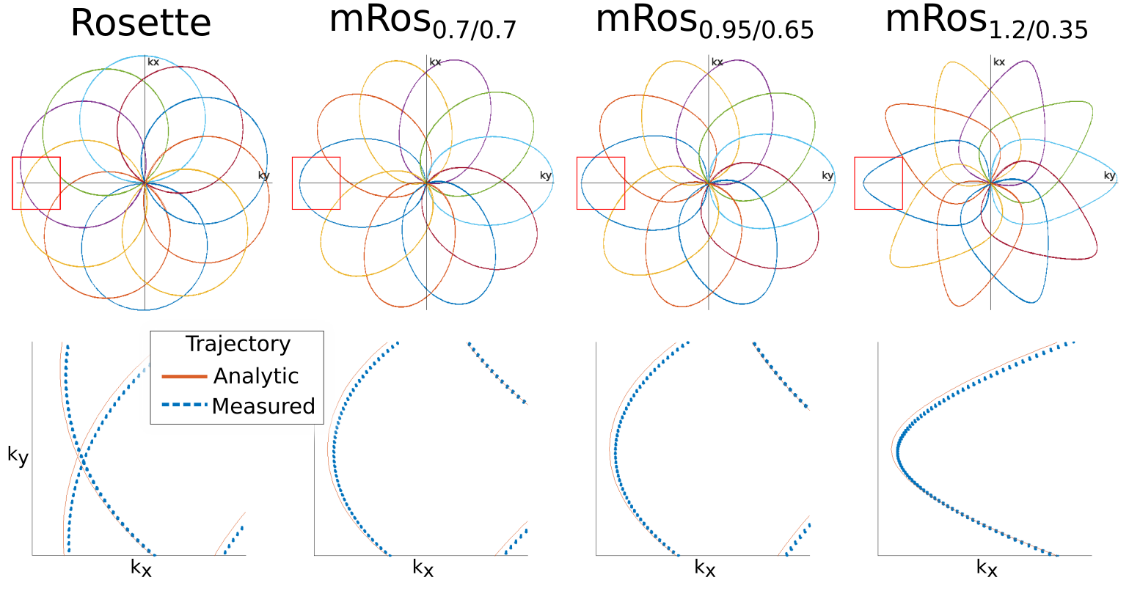


**Figure S1:** Distribution of petals in k-space of the sequences compared (top row). In the bottom row the analytic and measured k-space trajectories can be observed for the petal with rotation angle $\alpha=\pi$ (red box in the top row).

**
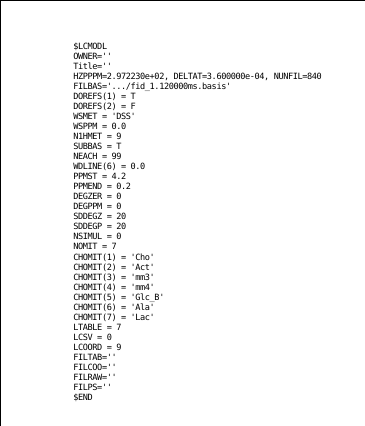
**

**Figure S2:** LCModel control file from an exemplary voxel.


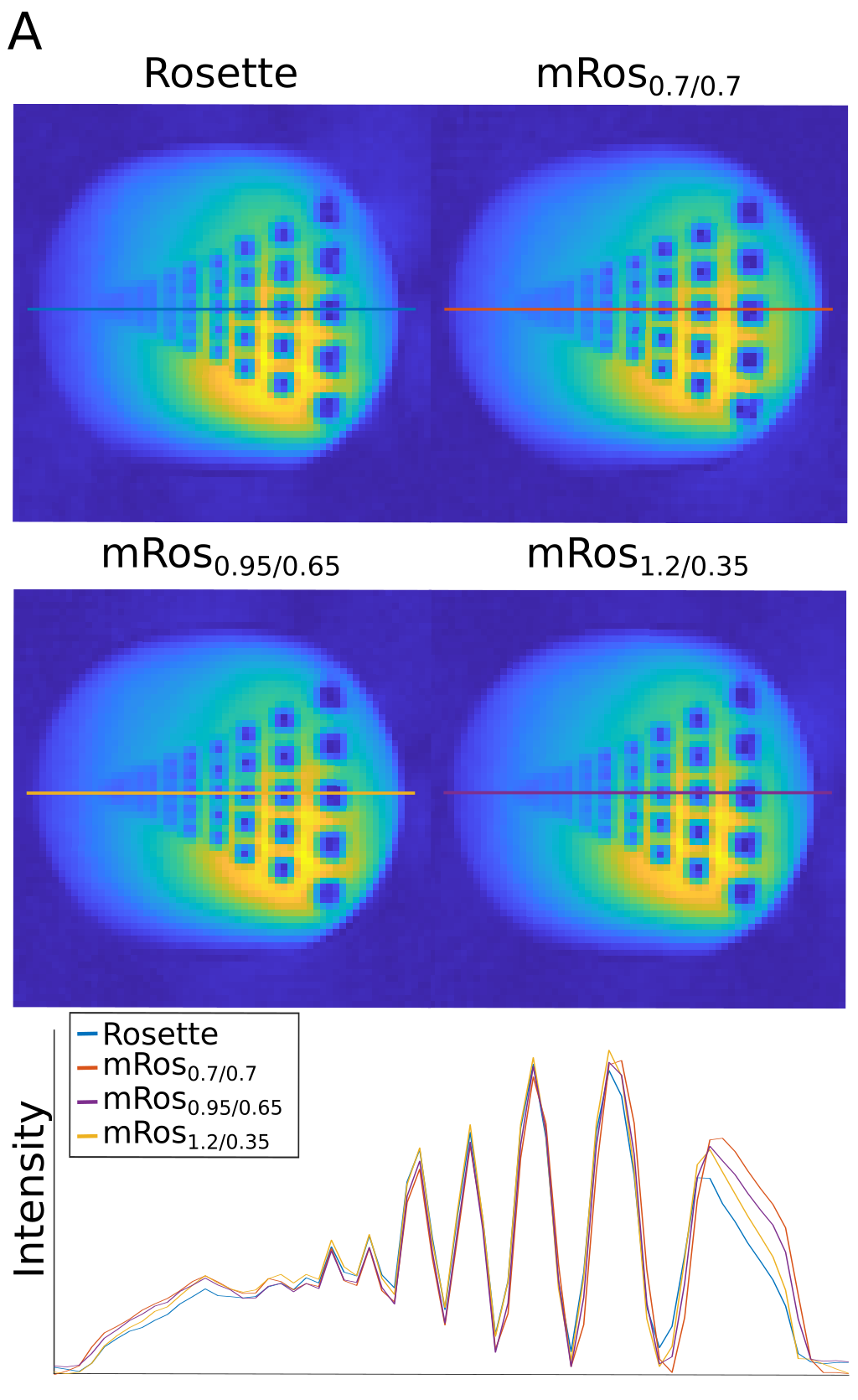


**Figure S3:** Resolution phantom experiments using the in vivo protocol. Pixel intensities along a horizontal line in the center of the image are shown below. Comparable resolution capabilities can be observed for all compared trajectories.


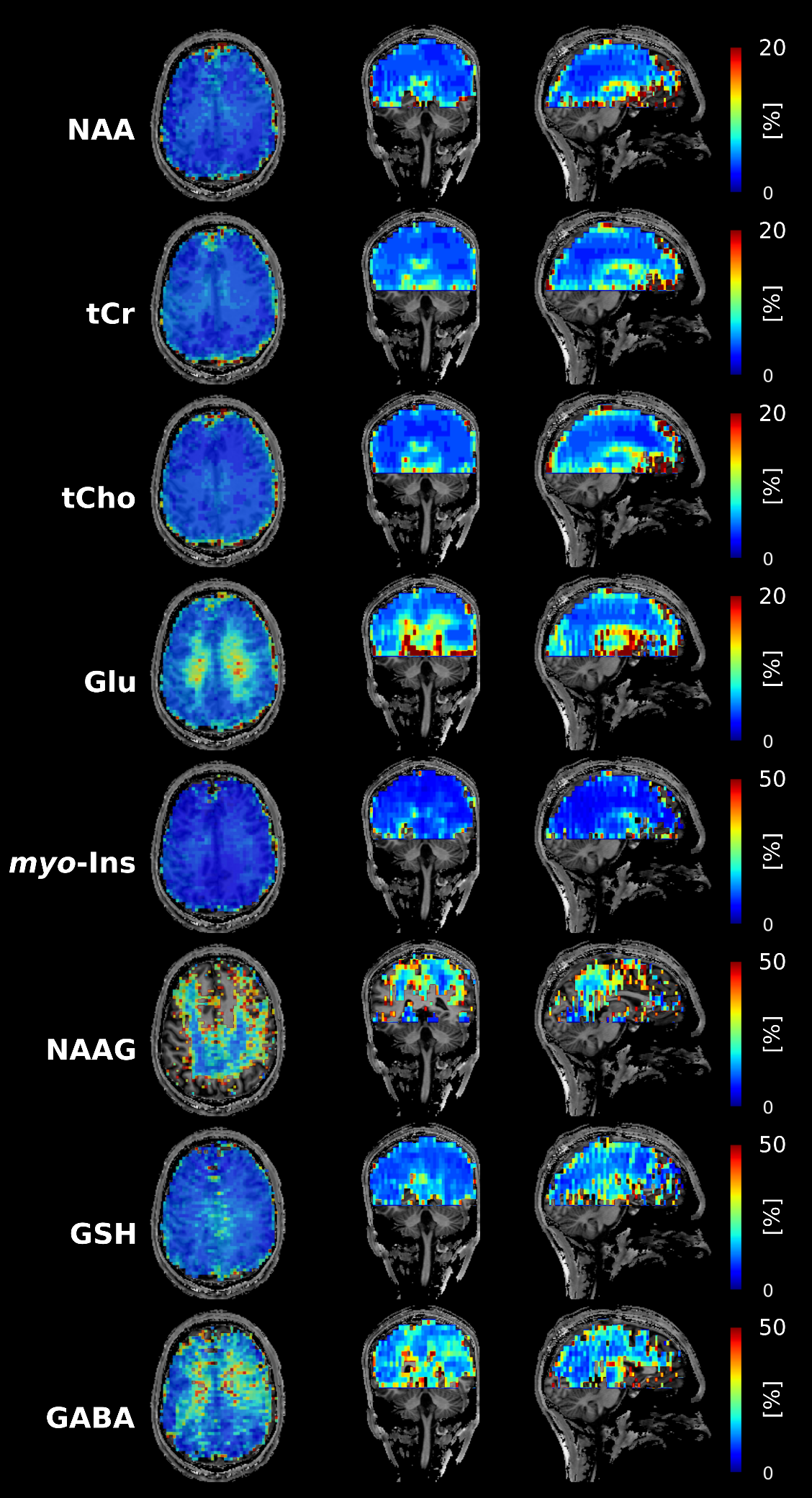


**Figure S4:** Whole-brain CRLB maps of the metabolites shown in Figure 7.


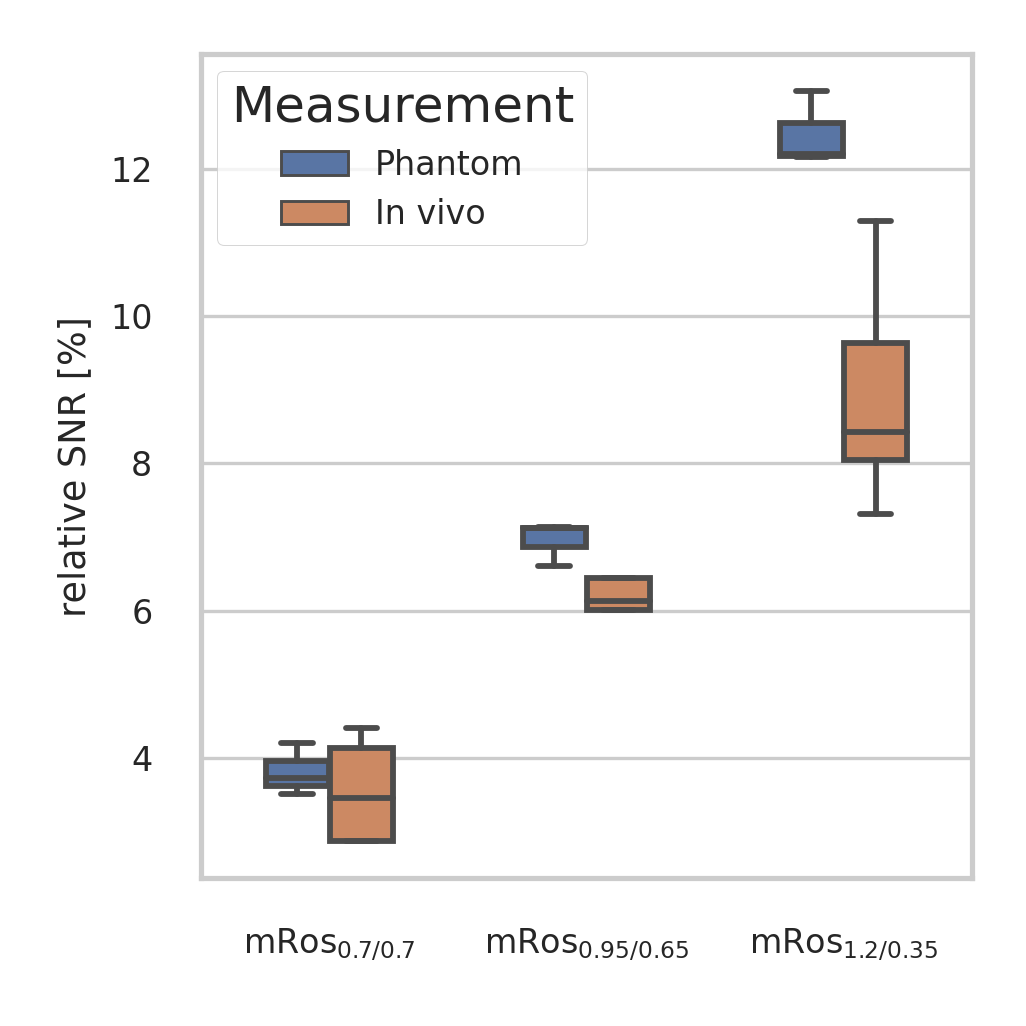


**Figure S5:** Boxplot showing the increase in SNR for the modified rosette trajectories compared to the rosette trajectory. The results for the phantom and in vivo measurements are shown.

**Table S1:** Minimum Reporting Standards for in vivo MR Spectroscopy Note. – Parameters 7T DMI, CRLB = Cramér-Rao lower bounds; FID = free induction decay; FOV = field of view; SNR = signal-to-noise ratio; VOI = volume of interest.

|  |  |  | |  |
| --- | --- | --- | --- | --- |
| *Minimum Reporting Standards in MR Spectroscopy checklist (according to Lin et al. NMR Biomed 2021)* | | | | |
| **1. Hardware** | | |  | |
| *a. Field strength [T]* | | | 7T | |
| *b. Manufacturer* | | | Siemens | |
| *c. Model (software version if available)* | | | Magnetom 7T Plus | |
| *d. RF coils: nuclei (transmit/ receive), number of channels, type, body part* | | | 32 channel ^1^H head coil, transmit/receive, 1 channel, (Nova Medical, Wilmingtom, MA) | |
| *e. Additional hardware* | | | N/A | |
| **2. Acquisition** | | |  | |
| *a. Pulse sequence* | | | 2D FID-acquire elliptical phase encoding (PE) MRSI,  2D FID-acquire rosette trajectory MRSI  2D FID-acquire egg-shaped petal MRSI  3D FID-acquire egg-shaped petal MRSI | |
| *b. Volume of Interest (VOI) locations* | | | Posterior cingulate gyrus | |
| *c. Nominal VOI size [cm^3^, mm^3^]* | | | PE MRSI: FOV 220x220x10 mm^3^  Rosette MRSI: FOV 220x220x10 mm^3^  2D egg-shaped petal MRSI: 220x220x10 mm^3^  3D egg-shaped petal MRSI: 220x220x70 mm^3^ | |
| *d. Repetition Time (TR), Echo Time (TE) [ms, s]* | | | PE MRSI: TR=440 ms, TE=1.1 ms  Rosette MRSI: TR=437 ms, TE= 1.12 ms  2D egg-shaped petal MRSI: TR=437 ms, TE= 1.12 ms  3D egg-shaped petal MRSI: TR=440 ms, TE= 0.92 ms | |
| *e. Total number of Excitations or acquisitions per spectrum* | | | PE MRSI: 1 averages (22:02 min:sec acquisition time)  Rosette MRSI: 4 averages (1:30 min:sec acquisition time)  2D egg-shaped petal MRSI: 4 averages (1:30 min:sec acquisition time)  3D egg-shaped petal MRSI: 1 averages (18:44 min:sec acquisition time) | |
| *In time series for kinetic studies* | | | N/A | |
| *i.         Number of Averaged spectra (NA) per time-point* | | | N/A | |
| *ii.       Averaging method (e.g. block-wise or moving average)* | | | N/A | |
| *iii.      Total number of spectra (acquired / in time-series)* | | | N/A | |
| *f. Additional sequence parameters (spectral width in Hz, number of spectral points, frequency offsets); If STEAM: Mixing Time TM; If MRSI: 2D or 3D, FOV in all directions, matrix size, acceleration factors* | | | PE MRSI: BW: 4200 Hz, 2048 spectral points, 64x64  Rosette MRSI: BW: 2780 Hz, 840 spectral points, 64x64  2D egg-shaped petal MRSI: 2780 Hz, 840 spectral points, 64x64  3D egg-shaped petal MRSI: 2780 Hz, 840 spectral points, 64x64x17 | |
| *g. Water Suppression Method* | | | WET | |
| *h. Shimming Method, reference peak, and thresholds for “acceptance of shim” chosen* | | | Standard shim + manual adjustment, ^1^H water peak < 40 Hz | |
| *i. Triggering or motion correction method (respiratory, peripheral, cardiac triggering, incl. device used and delays)* | | | - | |
| **3. Data analysis methods and outputs** | | |  | |
| *a. Analysis software* | | | LCModel 6.3-1 | |
| *b. Processing steps deviating from quoted reference or product* | | | N/A | |
| *c. Output measure (e.g. absolute concentration, institutional units, ratio)* | | | concentration estimation in mM | |
| *d. Quantification references and assumptions, fitting model assumptions* | | | Simulated in NMRScope-B | |
| **4. Data Quality** | | |  | |
| *a. Reported variables (SNR, Linewidth (with reference peaks))* | | | SNR was calculated using the pseudo replica method | |
| *b. Data exclusion criteria* | | | No subjects excluded | |
| *c. Quality measures of postprocessing Model fitting (e.g. CRLB, goodness of fit, SD of residual)* | | | CRLB | |
| *d. Sample Spectrum* | | | See Figure 4 | |

**Table S2:** Results of the SNR per-unit-time calculation for the rosette trajectory and modified rosette trajectories from the phantom measurement. SNR values are displayed as the percentage difference from the rosette trajectory. The mean and standard error over all three measurements are shown.

|  | **mRos_0.7/0.7_** | **mRos_0.95/0.65_** | **mRos_1.2/0.35_** |
| --- | --- | --- | --- |
| **Meas 1** | 3.51 | 7.14 | 12.20 |
| **Meas 2** | 3.73 | 6.61 | 12.16 |
| **Meas 3** | 4.20 | 7.12 | 13.05 |
| **Mean** | 3.81± 0.20 | 6.96± 0.17 | 12.47± 0.29 |
